# Gut microbiota response to *Enterocytozoon bieneusi* infection in wild rodents: enhanced vitamin B and K_2_ biosynthesis pathways

**DOI:** 10.1186/s12864-026-12575-4

**Published:** 2026-02-05

**Authors:** Xiao-Xuan Zhang, He Zhang, Ji-Xin Zhao, Hai-Long Yu, Chun-Ren Wang, Kai-Meng Shang, Yong-Jie Wei, Ya Qin, Jian-Ming Li, Zi-Yu Zhao, Chang-You Xia, Bei-Ni Chen, Hany M. Elsheikha, He Ma

**Affiliations:** 1 College of Veterinary Medicine, Qingdao Agricultural University, Qingdao, Shandong Province People’s Republic of China; 2 State Key Laboratory of Animal Disease Control and Prevention, Harbin Veterinary Research Institute, Chinese Academy of Agricultural Sciences, Harbin, Heilongjiang Province People’s Republic of China; 3 College of Veterinary Medicine, Jilin Agricultural University, Changchun, Jilin Province People’s Republic of China; 4 College of Animal Science and Veterinary Medicine, Heilongjiang Bayi Agricultural University, Daqing, Heilongjiang Province People’s Republic of China; 5 College of Chinese Medicinal Materials, Jilin Agricultural University, Changchun, Jilin Province People’s Republic of China; 6 Jilin Provincial Engineering Research Center for Efficient Breeding and Product Development of Sika Deer, Changchun, Jilin Province People’s Republic of China; 7 Key Laboratory of Animal Production and Product Quality and Security, Ministry of Education, Ministry of National Education, Changchun, Jilin Province People’s Republic of China; 8 College of Life Sciences, Changchun Sci-Tech University, Shuangyang, Jilin Province People’s Republic of China; 9 Faculty of Medicine and Health Sciences, School of Veterinary Medicine and Science, University of Nottingham, Sutton Bonington Campus, Loughborough, UK

**Keywords:** *Enterocytozoon bieneusi*, Wild Rodents, Gut Microbiota, Host-Microbe Interactions, Vitamin Synthesis, Comparative Genomics

## Abstract

*Enterocytozoon bieneusi* (*E. bieneusi*) is a pathogenic microsporidian that affects immunocompromised individuals, including those with HIV, and represents a major cause of diarrhea. It can severely impact human health, causing gastrointestinal disease, nutritional deficits, and life-threatening complications. However, the microbial mechanisms by which *E. bieneusi* affects host nutrition are not well understood. Wild rodents have long been considered valuable models for studying human diseases due to similarities in gut microbiota dynamics and immune responses, making them particularly relevant for investigating parasitic infections. Here, we assembled a comprehensive catalog of 9,929 non-redundant microbial genomes from wild rodent gut metagenomes and evaluated their potential for B vitamins and vitamin K_2_ biosynthesis using comparative functional genomics. We identified 2,307 genomes encoding complete pathways for de novo biosynthesis of at least one essential vitamin, though no single genome encoded all pathways, indicating a distributed metabolic capacity within the microbial community. Infection with *E. bieneusi* significantly altered the microbial composition and the potential for vitamin biosynthesis, with a notable expansion of *Methanobacteriota* and reprogramming of pyridoxine (vitamin B_6_) biosynthesis pathways. These changes reveal a functional shift in microbial metabolism in response to parasitic pressure. By elucidating the microbial basis of vitamin biosynthesis in wild rodents and the impact of *E. bieneusi* infection on microbial functions, this study provides new insights into the role of gut microbiota in maintaining host health and supporting nutrient provision under parasitic stress. Moreover, the findings will provide valuable insights into the prevention and control of *E. bieneusi* infection in a variety of host, including humans.

**Supplementary Information:**

The online version contains supplementary material available at 10.1186/s12864-026-12575-4.

## Introduction

The gut microbiome plays a fundamental role in host metabolism and health. Previous studies have demonstrated that gut microbiota can synthesize substantial proportions of the host's B vitamin requirements, with vitamin K_2_ being almost exclusively microbial in origin [1, 2]. This microbial contribution is particularly important in wild animals, such as rodents, which do not receive dietary vitamin supplementation and depend largely on their gut microbiota for micronutrient acquisition [3, 4]. Wild rodents, inhabiting diverse ecological niches and feeding on variable natural diets [3, 5, 6], represent an ecologically relevant model for studying microbiota-driven nutrient synthesis under fluctuating environmental conditions. Furthermore, the gut microbiome of wild rodents exhibits diversity and dynamic changes similar to those of humans, influenced by social interactions and environmental pressures, making them a valuable model for studying human diseases [7–9].

However, the functionality of this microbial ecosystem can be disrupted by pathogenic infections, with potential consequences for host nutrient acquisition. *Enterocytozoon bieneusi* (*E. bieneusi*), an obligate intracellular parasite of the phylum *Microsporidia*, frequently infects the intestinal epithelium of wild rodents and other mammals, including humans [10–12]. As an important zoonotic pathogen, *E. bieneusi* opportunistically infects immunocompromised individuals, particularly HIV-infected patients, and is a leading cause of diarrhea in this population [13]. A recent meta-analysis estimated the overall prevalence of *E. bieneusi* infection in humans at 7.9% [14], while in certain populations with reported animal contact, seroprevalence can reach as high as 33% [7].

In both wildlife and humans, *E. bieneusi* infection is associated with impaired gut barrier integrity, dysbiosis, and reduced nutrient absorption [15, 16]. Although *E. bieneusi* is widely present and poses a zoonotic risk [17, 18], the impact of *E. bieneusi* infection on the gut microbiota’s functional role—particularly in the biosynthesis of B vitamins and vitamin K—remains poorly characterized. This gap is concerning given that micronutrient deficiencies can exacerbate infection severity, compromise host survival, and affect reproductive fitness—factors with important implications for both ecological dynamics and public health.

To address this knowledge gap, we investigate how gut microbial communities contribute to vitamin biosynthesis in wild rodents under both healthy and pathogen-challenged conditions. In this study, we systematically characterize the capacity of the wild rodent gut microbiota to synthesize B vitamins and vitamin K_2_ and assess how this functional potential is altered during *E. bieneusi* infection. By leveraging a comprehensive metagenomic dataset comprising 17,137 microbial genomes and integrating taxonomic and functional analyses, we provide new insights into the ecological and metabolic resilience of wild rodent microbiomes. This work advances our understanding of environmentally shaped microbiome functionality and highlights the metabolic vulnerabilities introduced by parasitic perturbation, providing insights that are relevant to ecosystem health, wildlife conservation, and zoonotic disease management.

## Methods

### Metagenome assembly and taxonomic classification of wild rodent gut genomes

A total of 17,137 wild rodent gut genomes were retrieved from the Figshare repository (10.6084/m9.figshare.28752050) [6]. The host origins of these genomes spanned multiple geographical regions and countries, including Austria, Brazil, China, Costa Rica, Germany, the Isle of May, Israel, Lithuania, Ukraine, and the United States. Taxonomic classification was performed using the GTDB-Tk v2.3.2 classify_wf workflow [19], based on the Genome Taxonomy Database (GTDB). To remove redundancy, strain-level de-duplication at 99% average nucleotide identity (ANI) was performed using dRep v3.4.3 [20] with the parameters ‘-pa 0.9 -sa 0.99 -nc 0.30 -cm larger –S_algorithm fastANI,’ resulting in 9,929 unique genomes. To further resolve species-level diversity, ANI was re-estimated among genomes sharing identical genus-level taxonomic classifications using dRep (v3.4.3) with the parameters ‘-pa 0.9 -sa 0.95 -nc 0.30 -cm larger –S_algorithm fastANI’ [21]. This analysis identified 5,312 species-level genome bins (SGBs). The phylogenetic tree generated by GTDB-Tk was visualized using iTOL v6.9.1 (https://itol.embl.de/).

### Functional analysis of vitamin-related microbial gene catalog

Open reading frames (ORFs) were predicted from 9,929 genomes using Prodigal v2.6.3 [22] with the parameter '-p single'. The resulting ORFs were clustered using MMseqs easy-cluster workflows [23] with the parameters: ‘–split-mode 2 –cov-mode 2 -c 0.9 –min-seq-id 0.95 –cluster-mode 2 –cluster-reassign 1’ [24]. This resulted in a non-redundant microbial gene catalog containing 260,273 genes, with redundancy reduced by clustering sequences sharing > 95% identity to ensure a unique representation of microbial gene diversity. Functional annotation was performed by comparing the clustered genes to the Kyoto Encyclopedia of Genes and Genomes (KEGG) database [25] using DIAMOND v2.1.8.162 [26] with the following parameters: ‘–min-score 60 –query-cover 70 –max-target-seqs 5 –masking 1.’ For each gene, the alignment with the highest bit score was used for functional classification. To identify genes involved in vitamin biosynthesis, we compiled a curated set of KEGG Orthologs (KOs) corresponding to canonical biosynthetic pathways for B vitamins and menaquinone, as defined in KEGG pathway maps. Genes were assigned to vitamin biosynthetic functions if their annotated KO matched one of the predefined pathway-specific functional roles.

### Phylogenetic, taxonomic, and functional analyses of 3,522 high-quality genomes

To ensure data quality, all genomes were re-evaluated using CheckM2 v1.0.1 [27], and only those with ≥ 90% completeness and < 5% contamination were retained. Following this filtering, strain-level de-redundancy at 99% ANI was performed using dRep v3.4.5 with the parameters ‘-pa 0.9 -sa 0.99 -nc 0.30 -cm larger –S_algorithm fastANI,’ resulting in 3,522 high-quality, non-redundant genomes. Functional annotation was conducted using DIAMOND v2.1.8.162 via BLASTP searches against the KEGG database. Essential functional roles for vitamin B and menaquinone biosynthesis were defined based on KO annotations, and a genome was considered capable of de novo biosynthesis if it contained all KOs required for at least one canonical pathway. Using these criteria, 2,307 genomes were predicted to synthesize vitamins B and menaquinone de novo.

### Host-specific de novo vitamin biosynthesis in gut microbiota

To compare de novo vitamin biosynthetic capacities of gut microbiota across different hosts, we collected genomes predicted to be capable of de novo vitamin biosynthesis in ruminants [1] and chickens [28] from previously published studies. In addition, the same analytical pipeline was applied to the comprehensive mouse microbiota genome (CMMG) catalog [29], resulting in the identification of 1,707 gut microbial genomes from laboratory mice predicted to possess de novo vitamin biosynthetic capabilities, using the same analytical pipeline.

### Effects of *E. bieneusi* infection on the gut microbiota

We reanalyzed metagenomic data from project PRJNA1175865, which comprises 20 gut metagenomic samples from wild rodents (Supplementary Table 1). As described in our previous study [30], the dataset includes 10 control (CON) and 10 *E. bieneusi*-infected samples. To ensure high data quality, raw reads were processed using fastp v0.23.0 [31] with the following parameters ‘-u 30 -n 5 -q 20 -y -Y 30 -l 80 –trim_poly_g’. Host-derived sequences were removed by aligning the quality-filtered reads to the rodent reference genome (NCBI RefSeq assembly: GCF_036323735.1) using Bowtie2 v2.5.0 [32]. The resulting clean reads were retained for downstream analyses. For functional profiling, clean reads were aligned to a non-redundant microbial gene catalog using Bowtie2 v2.5.0. as a reference, we assigned metagenomic reads to the respective groups with Bowtie2 v2.5.0. Read counts were normalized to reads per kilobase per million mapped reads (RPKM) to account for gene length and sequencing depth. The relative abundances of KOs were calculated by summing the RPKM values of genes assigned to each KO. For taxonomic profiling, taxon-level relative abundances were estimated within a genome-resolved framework based on GTDB taxonomy by aggregating the RPKM values of all genes (or genomes) assigned to each taxonomic unit [1, 28, 33]. Functional roles related to vitamin biosynthesis were defined based on KO annotations, and their relative abundances were calculated by summing the RPKM values associated with each role.

### Statistical analyses and visualization

Statistical analyses were conducted using R version 4.2.2. Alpha diversity metrices, including Shannon diversity and richness indices, were calculated for each sample based on both taxonomic and functional gene abundance data. Beta diversity was evaluated using Principal Coordinate Analysis (PCoA) based on Bray–Curtis dissimilarity, with group differences evaluated using permutational multivariate analysis of variance (PERMANOVA). The Wilcoxon rank-sum test was applied to identify significant differences in diversity indices and the relative abundance of taxa and functional features between groups. Rarefaction curves were generated using the ‘vegan’ package (v2.6–4). Chord diagrams were generated with the ‘circlize’ package (v2.8.0), and Sankey plots were constructed using ‘networkD3’ (v4.2.3). Network graphs were visualized using Gephi (v0.10.1). Additional plots were generated using the ‘ggplot2’ package in R.

## Results

### Construction and taxonomic profiling of a wild rodent gut microbiome genome catalog

**Fig. 1:**
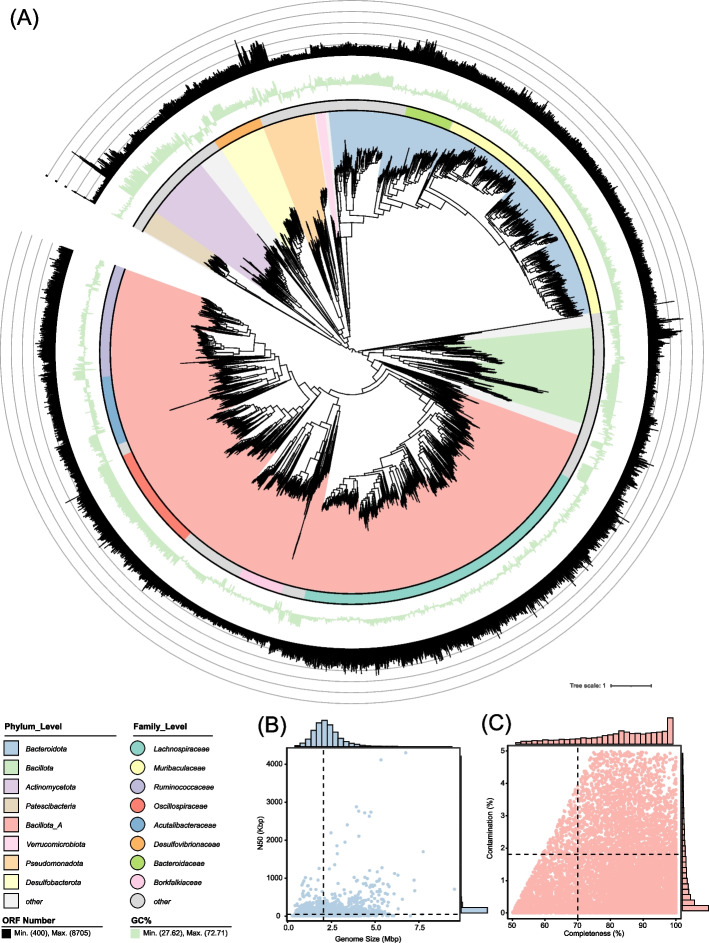
Phylogenetic and genomic characteristics of 5,312 species-level genomes. **A** Phylogenetic relationships among 5,312 species-level genomes, with each clade color-coded according to its phylum-level classification. From the inside to the outside, the first outer ring represents genus-level classification, the second outer ring depicts GC content, and the third outer ring indicates the number of ORFs in each genome. **B** Distribution of N50 values and genome sizes. **C** Assessment of genome completeness and contamination rates

To characterize the gut microbiota of wild rodents, we constructed a bacterial genome catalog by integrating 17,137 publicly available genomes, including 16,856 metagenome-assembled genomes (MAGs) and 281 genomes from cultured isolates. Genomes were filtered based on quality criteria (≥ 50% completeness, < 5% contamination, and [completeness – (5 × contamination)] ≥ 50) and dereplicated at a 99% ANI threshold. This process yielded 9,929 non-redundant genomes for downstream analysis (Supplementary Table 2). These genomes ranged in size from 0.26 Mb to 9.54 Mb (mean: 2.15 Mb), with an average N50 of 55,631 bp, 82.77% completeness, and 1.18% contamination (Fig. 1B-C). Interestingly, 3,522 genomes met high-quality standards (completeness ≥ 90%, contamination < 5%), providing a robust dataset for investigating microbial functional diversity.

To assess species-level diversity, we clustered the genomes at a 95% ANI threshold, resulting in 5,312 SGBs. These genomes had an average GC content of 48.54% (range: 22.21%–73.68%) and contained, on average, 2,074 ORFs per genome (range: 400–8,705). Taxonomic classification using GTDB-Tk database assigned the genomes to 24 phyla, 31 classes, 78 orders, 164 families, and 712 genera. At the phylum level, *Bacillota_A* (*n* = 2,731) and *Bacteroidota* (*n* = 1,312) were most abundant, followed by *Bacillota* (*n* = 354), *Actinomycetota* (*n* = 241), and *Pseudomonadota* (*n* = 195). At the family level, *Lachnospiraceae* (*n* = 1,069) and *Muribaculaceae* (*n* = 862) were most common, followed by *Ruminococcaceae (n* = 393), *Oscillospiraceae* (*n* = 358), and *Acutalibacteraceae* (*n* = 236) (Fig. 1A). Together, these genomes form a high-resolution reference dataset for exploring microbial diversity and function in wild rodents, including the genomic basis for traits such as vitamin biosynthesis that may contribute to host adaptation in dynamic environments.

### Identification and characterization of vitamin synthesis genes in the wild rodent gut microbiome

Using the 9,929 high-quality genomes described above, we investigated the potential for vitamin synthesis within the wild rodent gut microbiota. Protein-coding genes from these genomes were annotated against the KEGG database to assess their functional roles. In total, 464,312 genes corresponding to 199 KOs were identified as being involved in the biosynthesis of eight vitamin B compounds (biotin, cobalamin, folate, niacin, pantothenic acid, pyridoxine, riboflavin, and thiamine) as well as menaquinone (Supplementary Table 3). These vitamins were selected for focused analysis because gut microbiota are known to synthesize most B vitamins required by the host, while menaquinone is almost exclusively of microbial origin. To reduce redundancy, these genes were clustered using MMseqs, resulting in a non-redundant catalog of 260,273 genes with an average length of 895 bp. This catalog offers a valuable resource for functional classification and exploration of gut microbiota-mediated vitamin synthesis in wild rodents. Further pathway analysis based on this gene set revealed that all eight vitamin B compounds are synthesized directly by the gut microbiota, whereas menaquinone synthesis proceeds via indirect microbial pathways (Supplementary Fig. 1–3). These findings highlight the significant metabolic contributions of the gut microbiome to host vitamin availability. The presence of diverse and complete biosynthetic pathways across multiple taxa underscores a distributed metabolic architecture that may help sustain micronutrient levels in the host, particularly under nutrient-limited conditions.

### Host-specific genomic potential for de novo vitamin biosynthesis in gut microbiomes

**Fig. 2:**
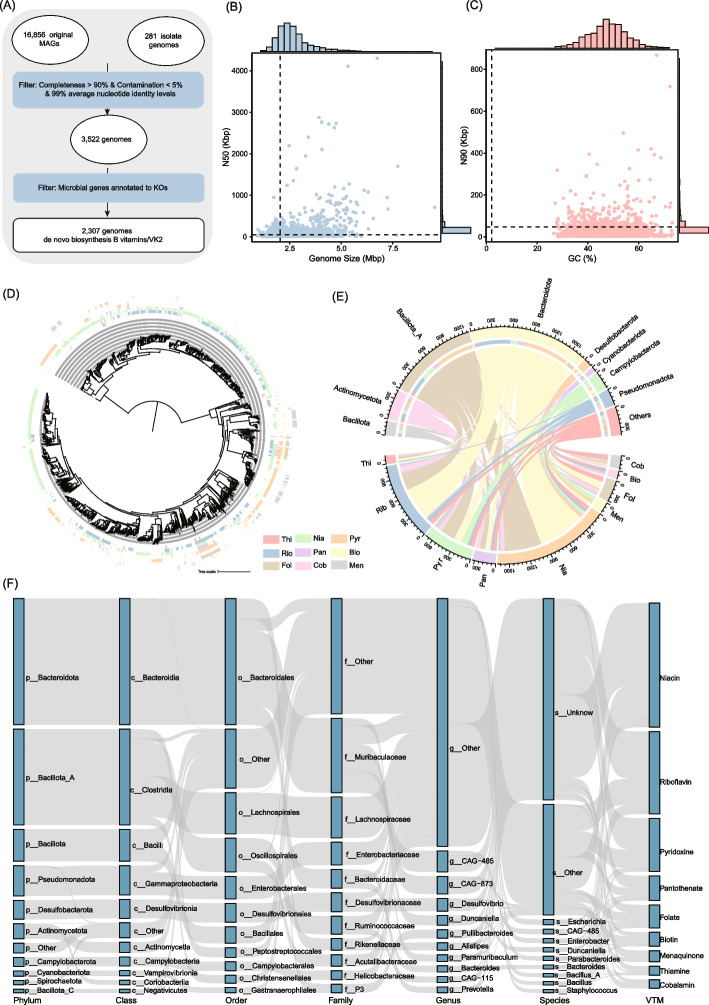
Genomic identification and phylogenetic distribution of vitamin synthesis potential.** A** Workflow for identifying genomes capable of synthesizing B and K_2_ vitamins. **B-C** Genomic statistics for 2,307 genomes. **D** Maximum-likelihood phylogenetic tree of 2,307 genomes. Outer-layer heatmaps indicate the presence (colored) or absence (blank) of vitamin synthesis capabilities. **E** Chord diagram showing the distribution of genomes with vitamin synthesis potential across different phyla. Each vitamin and phylum is represented by a distinct color. **F** Sankey diagram illustrating the relationships between taxonomic levels (phylum, class, order, family, and genus) and vitamin types

Building upon the curated gene sets for vitamin synthesis, we investigated the genome-level potential for de novo synthesis of B vitamins and menaquinone in the gut microbiome of wild rodents. From the 9,929 high-quality MAGs, we selected 3,522 with ≥ 90% completeness and < 5% contamination for downstream analysis (Supplementary Table 4). Functional annotation revealed that 2,307 genomes encoded complete pathways for synthesizing at least one B vitamin or menaquinone (Fig. 2A and Supplementary Table 5), signifying widespread but uneven biosynthetic potential across the microbiome. These genomes exhibited broad genomic diversity, with sizes ranging from 0.97 to 9.54 Mbp (mean = 2.64 Mbp), average N50 of 103,843 bp, and GC content spanning 26.58% to 73.42% (mean = 48.19%) (Fig. 2B-C). Taxonomic analysis showed that *Bacteroidota* (37.06%,, 855 genomes) and *Bacillota_A* (36.11%, 833 genomes) predominated among vitamin-producing genomes, followed by *Bacillota* (6.07%, 140 genomes), *Desulfobacterota* (5.21%, 118 genomes), and *Pseudomonadota* (3.80%, 90 genomes). This taxonomic distribution highlights key bacterial lineages contributing to gut-derived micronutrient synthesis in wild rodents.

Functional analysis of vitamin synthesis capabilities revealed that 961 genomes encoded the capacity to synthesize a single vitamin, while 1,314 were capable of producing 2 to 6 vitamins. Interestingly, 32 genomes were equipped to produce 7 or 8 vitamins, but none harbored complete biosynthetic pathways for all nine, underscoring the metabolic interdependence among microbial taxa and the likely necessity of cross-feeding in vitamin provisioning (Fig. 2D and Supplementary Table 5**)**. Among the biosynthetic targets, the most commonly encoded pathways were for niacin (1,664 genomes), riboflavin (1,106), and pyridoxine (713), with fewer genomes able to synthesize pantothenate, folate, biotin, thiamine, and cobalamin (Fig. 2E). The distribution of biosynthetic capacity was taxon-specific. *Bacteroidota* and *Bacillota_A* were the dominant phyla facilitating vitamin biosynthesis in wild rodents. Niacin, riboflavin, pyridoxine, and pantothenate were primarily synthesized by *Bacteroidota* (44.83%, 48.10%, 17.95%, and 25.45%, respectively) and *Bacillota_A* (35.52%, 25.86%, 32.96%, and 25.45%, respectively). Folate synthesis was mostly mediated by *Bacteroidota* (38.76%) and *Pseudomonadota* (23.17%). Biotin synthesis was mainly supported by *Pseudomonadota* (38.17%) and *Campylobacterota* (16.67%). Menaquinone synthesis was dominated by *Desulfobacterota* (45.33%) and *Campylobacterota* (31.33%). Thiamine synthesis was chiefly attributed to *Bacillota_A* (32.77%) and *Bacillota* (21.85%). Cobalamin synthesis was more evenly split between *Bacillota* (19.66%) and *Pseudomonadota* (19.66%) (Fig. 2F).

To explore whether this biosynthetic landscape was conserved across host species, we extended our analysis to 1,707 MAGs from the laboratory mouse gut microbiome. Comparative profiling revealed distinct host-specific taxonomic patterns in vitamin synthesis. In cats, the predominant contributors were *Bacillota_A* (40.54%) and *Actinobacteria* (20.84%), whereas in ruminants, *Bacteroidota* (43.28%) and *Bacillota* (38.25%) were most prominent. In chickens, vitamin synthesis was primarily associated with *Bacteroidota* (28.21%) and *Bacillota_A* (25.66%). Although *Bacteroidota* and *Bacillota_A* were dominant in both wild rodents and laboratory mice, their relative contributions shifted—*Bacteroidota* led in wild rodents (37.06%), while *Bacillota_A* dominated in laboratory mice (52.9%) (Supplementary Fig. 4). These host-specific microbial vitamin profiles underscore variation in the taxonomic architecture of vitamin biosynthesis, reflecting differences in gut microbial composition between species.

### Impact of *E. bieneusi* infection on vitamin biosynthesis by the gut microbiota in wild rodents

**Fig. 3:**
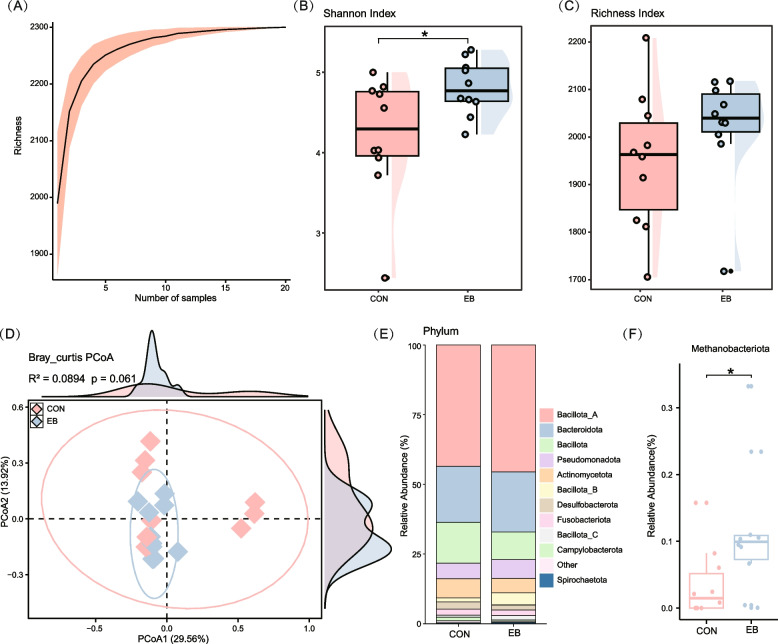
Diversity and taxonomic distribution of microbial genomes involved in vitamin B and K_2_ biosynthesis. **A** Rarefaction curve analysis illustrating the relationship between genome accumulation and increasing sample size. **B-C** Raincloud plots combining dot plots, boxplots, and distribution plots. The distribution plot represents probability density, the dot plot visualizes sample data point distribution, and the boxplot displays the richness and Shannon index of microbial genomes associated with vitamin B and K_2_ biosynthesis. Statistical significance was determined using the Wilcoxon rank sum test: * *p* < 0.05; ** *p* < 0.01; *** *p* < 0.001. **D** Scatter plots depicting beta diversity, highlighting compositional changes in microbial genomes related to vitamin B and K_2_ biosynthesis. Samples are plotted based on the first and second principal coordinates (PCoA1 and PCoA2), with explained variance percentages indicated. Ellipsoids represent the 95% confidence interval for each group. Line graphs above and to the right illustrate sample distribution along PCoA1 and PCoA2, reflecting density variations between groups. **E** Bar graph showing the phylum-level taxonomic distribution of gene sets associated with vitamin B and K_2_ biosynthesis. **F** Boxplots showing the differences in the relative abundance of *Methanobacteriota* before and after *E. bieneusi* infection. Statistical significance was assessed using the Wilcoxon rank-sum test (*p < 0.05)

Having established the capacity of wild rodent gut microbiota for de novo vitamin biosynthesis, we next investigated how *E. bieneusi* infection alters this functional potential. Although gut microbes are central to host vitamin metabolism, the effects of parasitic infection on microbial vitamin-producing capacity remain poorly characterized. To address this, we reanalyzed gut metagenomic data from infected and uninfected rodents, focusing on genomes annotated with vitamin biosynthetic pathways. Rarefaction analysis confirmed sufficient sequencing depth to capture the diversity of vitamin-producing genomes, with cumulative curves reaching saturation (Fig. 3A).

Alpha diversity analysis revealed a significant increase in the Shannon index of vitamin-synthesizing genomes in infected rodents, indicating enhanced within-sample diversity post-infection (Fig. 3B–C). Beta diversity assessed via PCoA explained 43.48% of the variation across the first two axes. Although a shift in community composition was observed between infected and uninfected groups, this difference did not reach statistical significance (PERMANOVA: R2 = 0.0894, *p* = 0.075) (Fig. 3D). In contrast to the reduced overall taxonomic diversity typically reported in parasitic infections [34, 35], this increase in the diversity of the vitamin-synthesizing gene pool likely reflects a mechanism of functional compensation or redundancy adopted by the microbiota to address the host's elevated metabolic demands [36].

To identify specific taxa driving these changes, we conducted a differential abundance analysis of vitamin-producing phyla. Importantly, *Methanobacteriota* showed a significant increase in relative abundance following *E. bieneusi* infection (Fig. 3E-F and Supplementary Fig. 5), while other phyla remained stable. Interestingly, *Methanobacteriota* contributed exclusively to pyridoxine (vitamin B_6_) synthesis and was not implicated in the biosynthesis of any of the other eight vitamins, suggesting a targeted functional enrichment. These findings indicate that *E. bieneusi* infection selectively enriches microbial taxa involved in specific vitamin biosynthetic pathways, particularly pyridoxine. This functional shift implies a potential microbial adaptation to parasitic stress, wherein vitamin B_6_ production may confer a survival or ecological advantage within the altered gut environment.

### Pyridoxine biosynthesis and its modulation by *E. bieneusi* infection in wild rodent gut microbiota

**Fig. 4:**
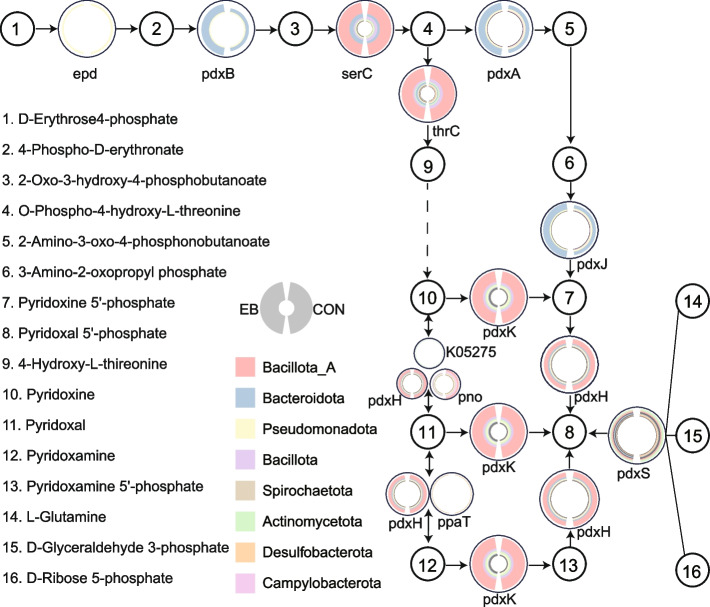
Phylogenetic distribution of genes involved in pyridoxine biosynthesis. Large circles represent functional roles in the de novo biosynthetic pathway of pyridoxine. Within each large circle, circular stacked bar charts depict the distribution of genes associated with these functional roles across different phylum-level classifications, with each phylum represented by a distinct color. Small circles indicate metabolites involved in the pyridoxine biosynthetic process

As part of our broader investigation into gut microbial vitamin production, we focused on pyridoxine, a coenzyme essential for amino acid metabolism, immune regulation, neurotransmitter synthesis, and environmental stress adaptation. Pyridoxine is synthesized by the gut microbiota and can, in turn, shape microbial community structure and function. We examined how *E. bieneusi* infection influences the genetic capacity for pyridoxine biosynthesis in the wild rodent gut. We identified two pyridoxine biosynthetic routes within the gut microbiota: a direct pathway catalyzed by pyridoxine 5′-phosphate synthase (involving Pdx proteins), and an alternative route using 4-hydroxy-L-threonine (4-HTL) as a precursor. Both pathways were constrained by the low relative abundance of the gene *epd* (CON: 0.006706954, EB: 0.005771136; Supplementary Table 6), which encodes erythrose-4-phosphate dehydrogenase, a key enzyme required for the biosynthesis of vitamin B6. Despite this bottleneck, *E. bieneusi* infection led to a moderate increase in the relative abundance of several pyridoxine-related genes, including *pdxB* (CON: 0.02579339, EB: 0.0505509), *pdxA* (CON: 0.0366146, EB: 0.04973373), *pdxJ* (CON: 0.0308526, EB: 0.0414727) and *pdxK* (CON: 0.2196965, EB: 0.222216). Core biosynthetic genes (*pdxB*, *pdxA*, *pdxJ*) were predominantly associated with the phylum *Bacteroidota*, whereas *epd* was enriched in *Pseudomonadota*. Auxiliary precursor-synthesis genes were largely contributed by *Bacillota_A* (Fig. 4). These data reveal a phylum-specific distribution of pyridoxine biosynthesis genes in the wild rodent gut microbiota. Following *E. bieneusi* infection, an increase in key biosynthetic genes suggests altered functional potential in vitamin B6 synthesis.

## Discussion

This study presents a comprehensive genomic and functional analysis of the gut microbiome in wild rodents, emphasizing its critical role in vitamin biosynthesis and the modulatory effects of *E. bieneusi* infection on microbial community structure and metabolic function. A key novel finding is the significant enrichment of pyridoxine producing *Methanobacteriota* in infected hosts, unveiling an adaptive microbial response to parasitic stress that has not been previously documented. By integrating publicly available datasets and implementing rigorous quality control measures, we assembled a curated collection of 9,929 high-quality bacterial genomes, providing a robust framework for future investigations into microbiome-mediated host–parasite interactions, functional diversity, and adaptations in fluctuating environments.

Our study uncovers a remarkable taxonomic richness within the wild rodent gut microbiota, comprising 5,312 SGBs across 24 bacterial phyla, underscoring the complexity of host-microbiome interactions in natural environments. The dominance of *Bacillota_A* and *Bacteroidota* aligns with their well-established roles in fiber degradation [37] and energy harvesting [38]. However, our data provide new insights into their relative contributions under wild dietary regimes. Importantly, members of *Lachnospiraceae* and *Muribaculaceae* emerge as important taxa critical for maintaining gut homeostasis through short-chain fatty acid (SCFA) production— metabolites fundamental for host energy balance and immune regulation [39, 40]. With 712 genera identified, this microbial consortium exhibits multifaceted functionality, mediating nutrient metabolism, immunomodulation, and environmental adaptability [41, 42]. Our findings highlight how such extensive microbial diversity equips wild rodents with a dynamic gut ecosystem capable of responding to fluctuating dietary inputs and ecological pressures, thereby shaping host physiology and resilience in natural habitats [4, 43–45].

Our data reveals that the wild rodent gut microbiome possesses extensive metabolic potential to support host vitamin nutrition, underscoring its functional importance beyond digestion. Functional annotation of 464,312 protein-coding genes identified 199 KOs involved in the biosynthesis of essential vitamins—including all eight B vitamins (biotin, cobalamin, folate, niacin, pantothenic acid, pyridoxine, riboflavin, and thiamine) as well as vitamin K₂. This comprehensive repertoire reflects a metabolically versatile microbiome capable of supplementing host micronutrient requirements, potentially alleviating dependence on dietary vitamin intake [46]. The detection of menaquinone synthesis pathways suggests complex syntrophic and cross-feeding interactions within the microbial community—ecological dynamics that enhance nutrient accessibility and stabilize microbial consortia [47]. These cooperative interactions exemplify the microbiome’s contribution to host metabolic homeostasis, immune development, and resilience [48, 49]. Moreover, inter-individual variations in vitamin biosynthesis capacity may modulate host fitness and susceptibility to disease under different environmental or nutritional pressures [50, 51].

The identification of diverse microbial vitamin biosynthesis pathways underscores the evolutionary co-adaptation of the gut microbiome to the host’s diet and ecological niche [43, 52]. Wild rodents, whose diets are predominantly plant-based and often low in bioavailable vitamins, face significant micronutrient constraints. Our findings suggest that the gut microbiota has functionally adapted to offset these limitations, providing a complementary source of essential vitamins and thereby reinforcing a mutualistic, co-evolved relationship with the host [53]. This microbial compensation likely reflects long-term selective pressures favoring hosts whose microbiomes can maintain nutrient provisioning under fluctuating dietary conditions. Such functional plasticity may enhance host survival and fitness in resource-variable environments, positioning the microbiome as a key adaptive partner in the evolutionary trajectory of wild rodents.

Our analysis of de novo vitamin biosynthesis in the wild rodent gut microbiome reveals a complex, distributed metabolic architecture involving diverse microbial taxa and cooperative synthesis strategies. Rather than relying on a single dominant species, vitamin production is partitioned across multiple microbial contributors—each encoding different components of biosynthetic pathways. This decentralized, community-level organization, not previously described in wild rodent systems [54], highlights the microbiome’s functional integration and its critical role in sustaining host vitamin homeostasis [1]. While dominant taxa orchestrate core biosynthetic processes, less-abundant phyla, such as *Desulfobacterota* and *Pseudomonadota*, play secondary roles, enhancing overall pathway completeness, metabolic flexibility, and niche specialization [55, 56]. This functional redundancy likely confers resilience to the system, enabling wild rodents to maintain micronutrient sufficiency even under dietary or environmental stress.

A critical insight from this study is that no single microbial genome within the gut microbiome encodes the complete biosynthetic machinery for all nine essential vitamins. Instead, vitamin synthesis is a cooperative function—shared among taxonomically and functionally diverse microbes. This underscores the gut microbiome’s role as an integrated metabolic network rather than a sum of isolated organisms [57]. Such interdependent biosynthesis networks suggest that evolutionary selection has favored microbial consortia capable of buffering the host against nutrient variability. However, this dependency also implies vulnerability: disruptions from dietary shifts, environmental stressors, or disease may destabilize cooperative functions, potentially leading to vitamin deficiencies [58, 59]. Understanding these microbial interdependencies is therefore crucial for predicting host resilience and guiding future interventions to preserve microbiome-mediated nutrition under ecological change.

Our comparative analysis of gut microbiomes across diverse host species—including wild rodents, laboratory mice, cats, ruminants, and chickens—reveals striking host-specific patterns in microbial vitamin biosynthesis. In wild rodents, *Bacteroidota* emerged as the dominant contributor to vitamin pathways, whereas *Bacillota_A* predominated in carnivorous hosts such as cats, reflecting the influence of host diet, gut morphology, and microbial adaptation to distinct ecological niches [60]. We observed marked divergence between the microbiomes of wild rodents and laboratory mice, particularly in the relative abundance and biosynthetic contributions of *Bacillota_A*. These differences raise important questions about the extent to which domestication, controlled housing conditions, and standardized diets shape microbiome composition and functional capacity [61]. The reduced ecological and dietary complexity in laboratory settings likely narrows microbial diversity and metabolic flexibility, with implications for host physiology and experimental outcomes. Our findings challenge the assumption that laboratory rodents adequately model natural host–microbiome dynamics. The metabolically versatile microbiomes of wild rodents, shaped by exposure to diverse environments and variable diets, may better reflect the adaptive potential of host-associated microbial communities [62, 63]. Incorporating wild-derived microbiome data into experimental frameworks could enhance the ecological validity and translational relevance of studies in nutrition, immunity, and disease modeling.

One of the most interesting findings from our study is the infection-induced enrichment of *Methanobacteriota* species with the genetic potential to synthesize pyridoxine. This observation not only reveals a novel aspect of microbial functional resilience under parasitic stress but also suggests that the microbiota may provide compensatory nutrients to mitigate the effects of infection on host health. Building on our characterization of the healthy microbiome's metabolic potential, we investigated how *E. bieneusi* infection perturbs these dynamics. *E. bieneusi* is known to disrupt intestinal barrier integrity, altering nutrient absorption and immune status—particularly in immunocompromised or juvenile hosts [64, 65]. These physiological disruptions may impose selective pressures on the gut microbiota, potentially leading to shifts in both community structure and function. The significant increase in the Alpha diversity of vitamin-synthesizing genomes following *E. bieneusi *infection suggests a functional reorganization in response to host metabolic stress. We interpret this as an ecological strategy of functional redundancy, whereby the enrichment of diversified producer strains (e.g., *Methanobacteriota*) compensates for the host's elevated vitamin demand under infection pressure, thus maintaining host-microbe homeostasis [36, 66]. We postulate that this specific functional resilience is driven by the fundamental dietary distinctions between wild and laboratory rodents. Unlike laboratory mice maintained on standardized, nutrient-rich chow, wild rodents consume highly variable, fiber-rich natural diets that are often seasonally limited in micronutrients [8]. High-fiber diets are known to promote the proliferation of methanogenic archaea (including *Methanobacteriota*), which serve as critical hydrogen sinks in the syntrophic fermentation of polysaccharides [67]. Consequently, the gut microbiome of wild rodents has likely co-evolved a robust capacity for de novo B-vitamin synthesis (such as pyridoxine) to buffer against these dietary fluctuations [68]. This pre-existing metabolic plasticity enables the rapid functional expansion we observed post-infection—a compensatory response that may be diminished or absent in laboratory animals raised under relaxed nutritional selection pressures [69].

Further analysis uncovered two distinct pyridoxine biosynthesis pathways (via Pdx proteins and 4-hydroxythreonine-4-phosphate dehydrogenase, 4-HTL), underscoring the metabolic versatility of the microbial community [70]. However, the observed limitation in *epd* gene abundance may constrain total pyridoxine production, suggesting partial—but not complete—compensatory capacity. Importantly, post-infection increase in abundance of key pyridoxine biosynthesis genes (*pdxA*, *pdxB*, *pdxJ* and *pdxK*) may suggest a targeted microbial response aimed at sustaining host immune function under parasitic stress [71]. This functional resilience, despite relatively stable community composition, provides compelling evidence for *E. bieneusi*-driven metabolic reprogramming within the gut microbiome. These adaptations may represent a microbiota-mediated buffering mechanism to preserve host-microbe equilibrium or, conversely, an opportunistic metabolic shift that facilitates parasite infection [72]. These findings highlight the dynamic nature of host-microbe-parasite interactions and underscore the potential for microbiota-targeted strategies to enhance infection resilience and support nutrient homeostasis during enteric parasitic challenges.

## Conclusions

This study presents a high-resolution genomic framework that reveals the complex taxonomic and functional architecture of wild rodent gut microbiomes. By uncovering the dynamic capacity of these microbial communities to adapt metabolically—particularly through the reprogramming of vitamin biosynthesis pathways in response to parasitic infection—we highlight the microbiome’s critical role in maintaining host physiological balance under ecological and pathogenic stress. The infection-induced increase in the abundance of pyridoxine synthesis exemplifies the functional resilience and cooperative potential of the gut microbiota, offering new insights into host–microbe co-adaptation. Importantly, our findings extend beyond ecological microbiology by offering a reference point for evaluating microbiome responses in natural versus controlled environments. They underscore the translational relevance of wild microbiome models for improving our understanding of microbial contributions to health, immunity, and disease tolerance. While further validation is needed, the functional adaptations observed here provide conceptual frameworks for understanding microbiome resilience and nutrient provision during infection.

## Supplementary Information

Supplementary Material 1.

Supplementary Material 2.

Supplementary Material 3.

## Data Availability

The wild gut microbial genomes analyzed in this study have been deposited in the Figshare repository (https://doi.org/10.6084/m9.figshare.28752050). The associated metagenomic samples, previously published, are accessible under accession numbers PRJNA1175865.
