# Supplementary material for: Gut microbiota response to Enterocytozoon bieneusi infection in wild rodents: enhanced vitamin B and K2 biosynthesis pathways
Source: BMC Genomics. 2026 Feb 5;27:258. doi: 10.1186/s12864-026-12575-4 (PMC12973552; doi:10.1186/s12864-026-12575-4)
Supplement: Supplementary file 2 — Supplementary Material 2. [file 12864_2026_12575_MOESM2_ESM.docx]

**Supplementary Information**

**Gut microbiota response to *Enterocytozoon bieneusi* infection in wild rodents: enhanced vitamin B and K_2_ pathways**

**Xiao-Xuan Zhang^1a^, He Zhang^2a^, Ji-Xin Zhao^1^, Hai-Long Yu^1,3^, Chun-Ren Wang^4^, Kai-Meng Shang^1^, Yong-Jie Wei^1^, Ya Qin^1,3^, Jian-Ming Li^5^, Zi-Yu Zhao^3^, Chang-You Xia^2*^, Bei-Ni Chen^6^, Hany M. Elsheikha^7*^ & He Ma^1*^**

1 College of Veterinary Medicine, Qingdao Agricultural University, Qingdao, Shandong Province, PR China.

2 State Key Laboratory for Animal Disease Control and Prevention, Harbin Veterinary Research Institute, Chinese Academy of Agricultural Sciences, Harbin, Heilongjiang Province, PR China.

3 College of Veterinary Medicine, Jilin Agricultural University, Changchun, Jilin Province, PR China.

4 College of Animal Science and Veterinary Medicine, Heilongjiang Bayi Agricultural University, Daqing, Heilongjiang Province, PR China.

5 College of Chinese Medicinal Materials, Jilin Agricultural University, Changchun, Jilin Province, PR China; Jilin Provincial Engineering Research Center for Efficient Breeding and Product Development of Sika Deer, Changchun, Jilin Province, PR China; Key Laboratory of Animal Production and Product Quality and Security, Ministry of Education, Ministry of National Education, Changchun, Jilin Province, PR China.

6 College of Life Sciences, Changchun Sci-Tech University, Shuangyang, Jilin Province, PR China.

7 Faculty of Medicine and Health Sciences, School of Veterinary Medicine and Science, University of Nottingham, Sutton Bonington Campus, Loughborough, United Kingdom.

a These authors contributed equally.

*E-mail: xiachangyou@caas.cn (Chang-You Xia); Hany.Elsheikha@nottingham.ac.uk (Hany M. Elsheikha); mahe@qau.edu.cn (He Ma)

**Supplementary Figures**

**
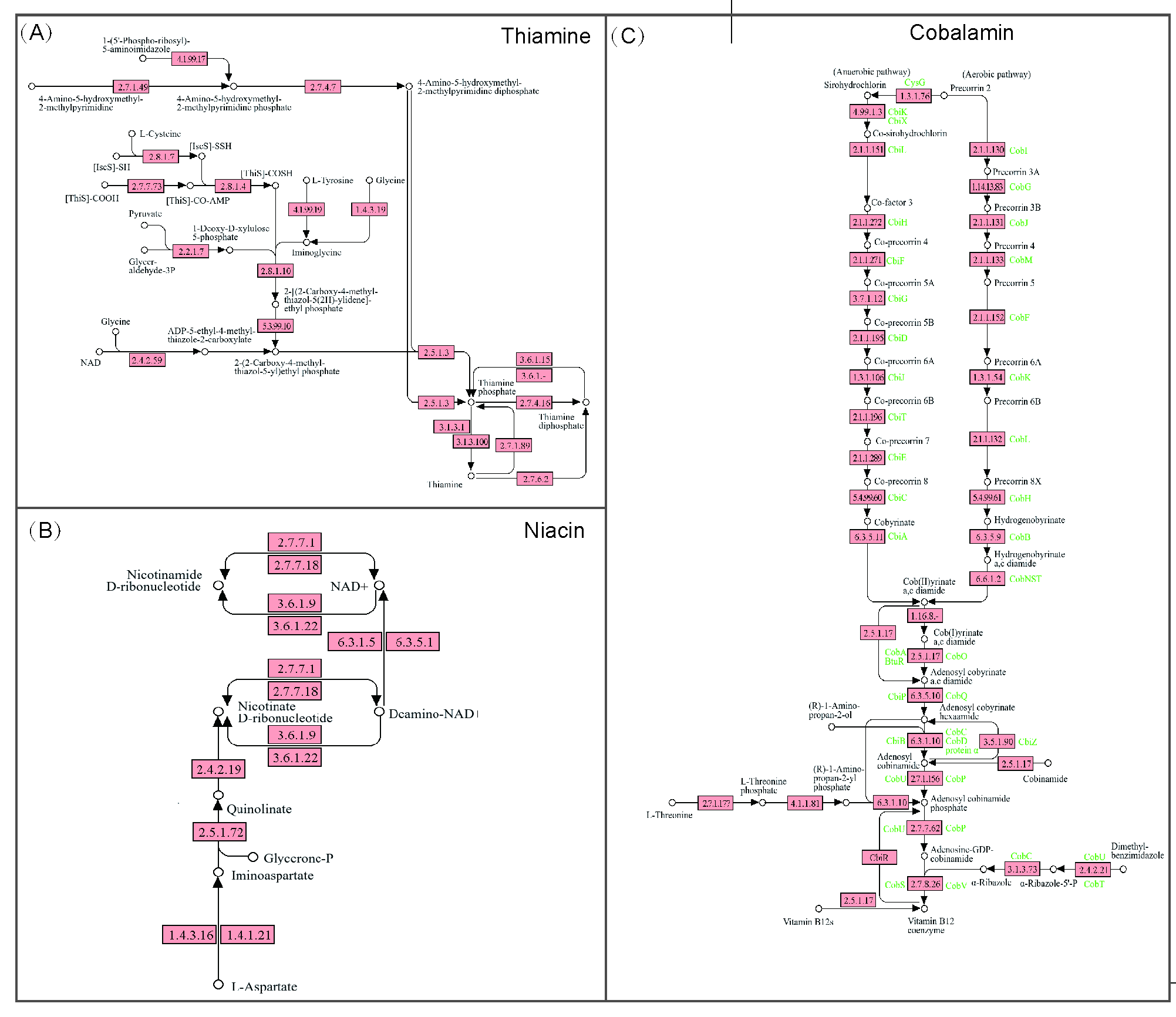
**

### **Supplementary Figure 1.** (A–C) Biosynthetic pathways of thiamine, niacin, and cobalamin. Functional roles are represented by rectangles, while metabolites are depicted as circles.


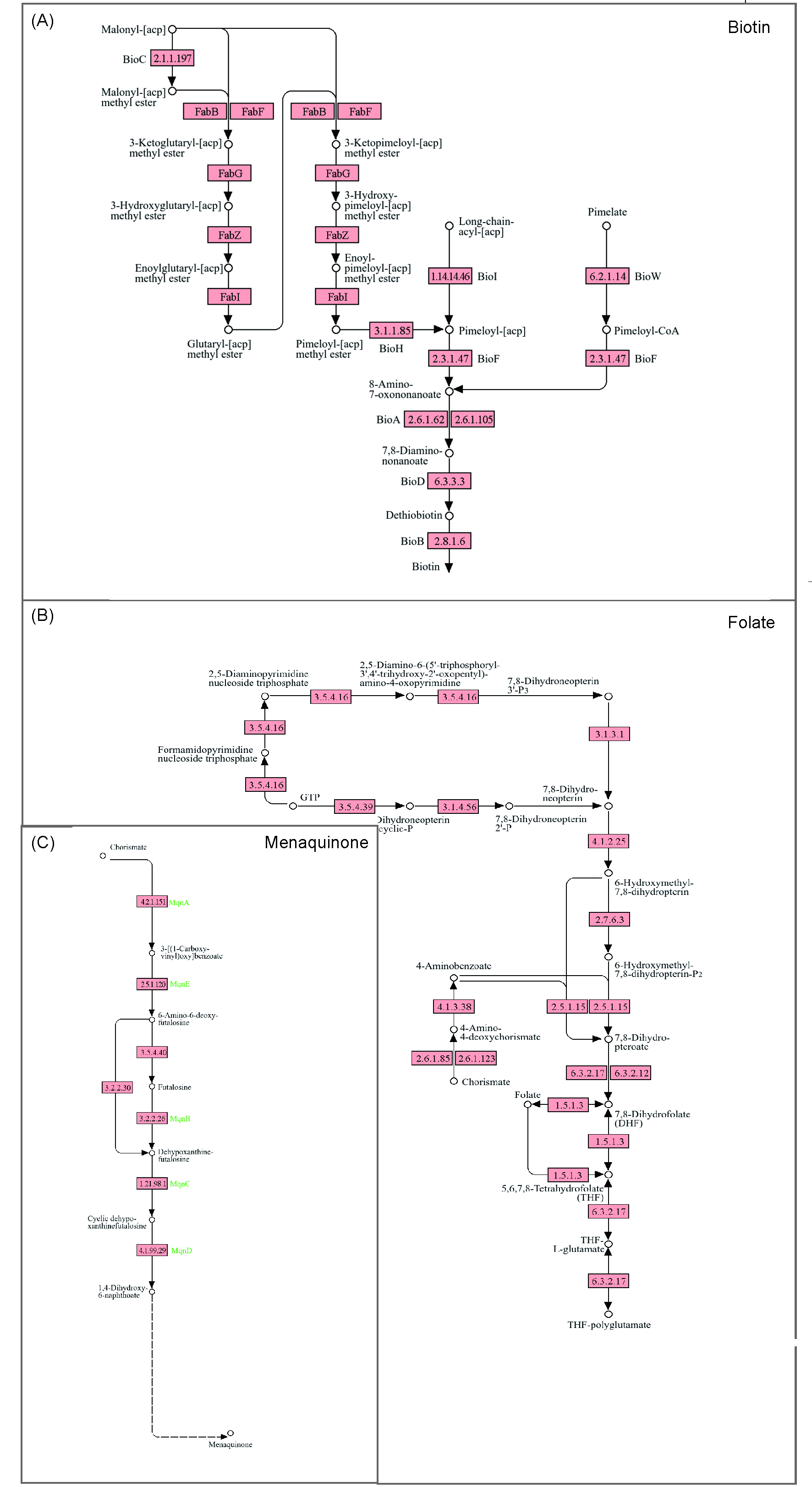


**Supplementary Figure 2.** (A–C) Biosynthetic pathways of biotin, folate, and menaquinone. Functional roles are represented by rectangles, while metabolites are depicted as circles.


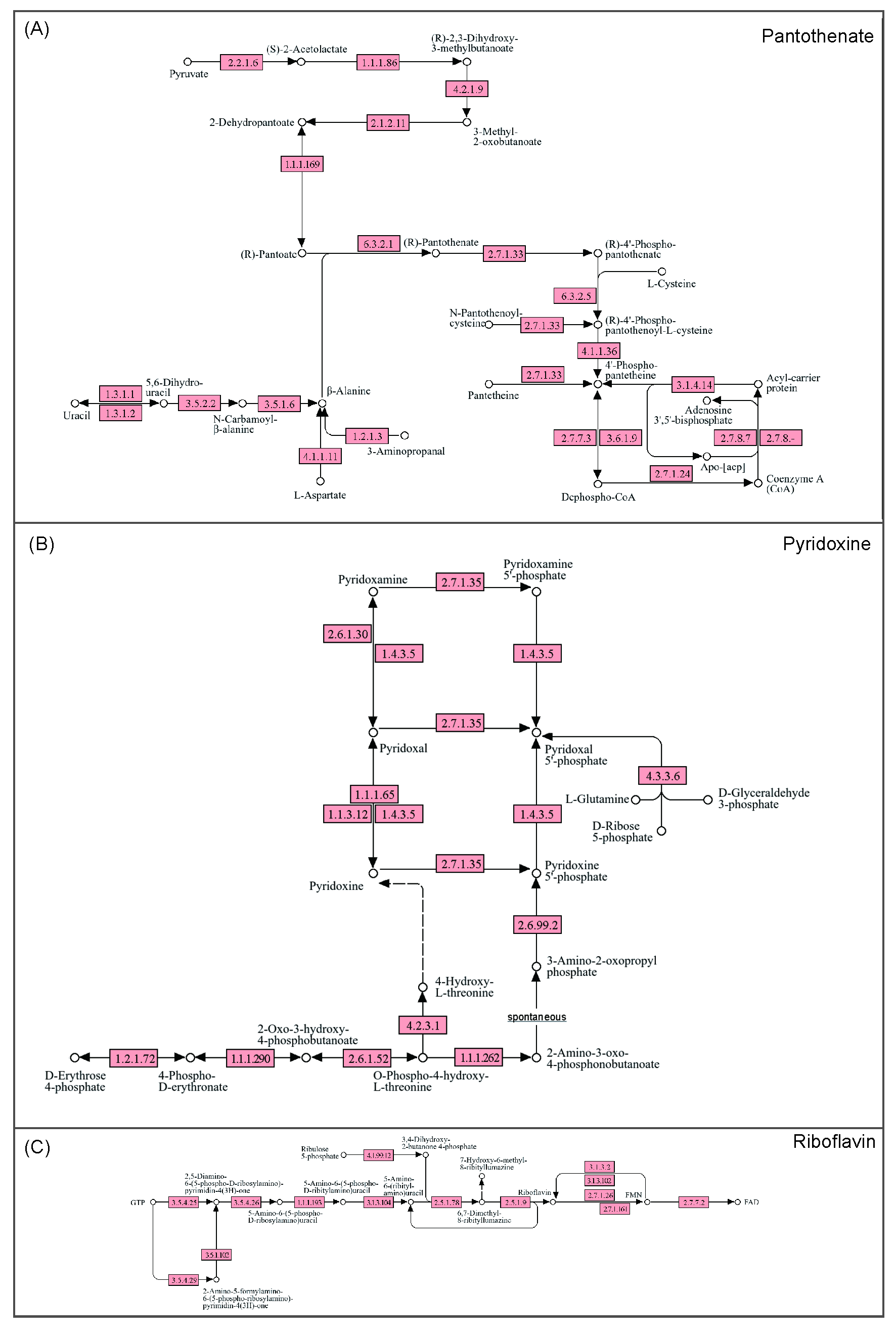


**Supplementary Figure 3.** (A–C) Biosynthetic pathways of pantothenate, pyridoxine, and riboflavin. Functional roles are represented by rectangles, while metabolites are depicted as circles.


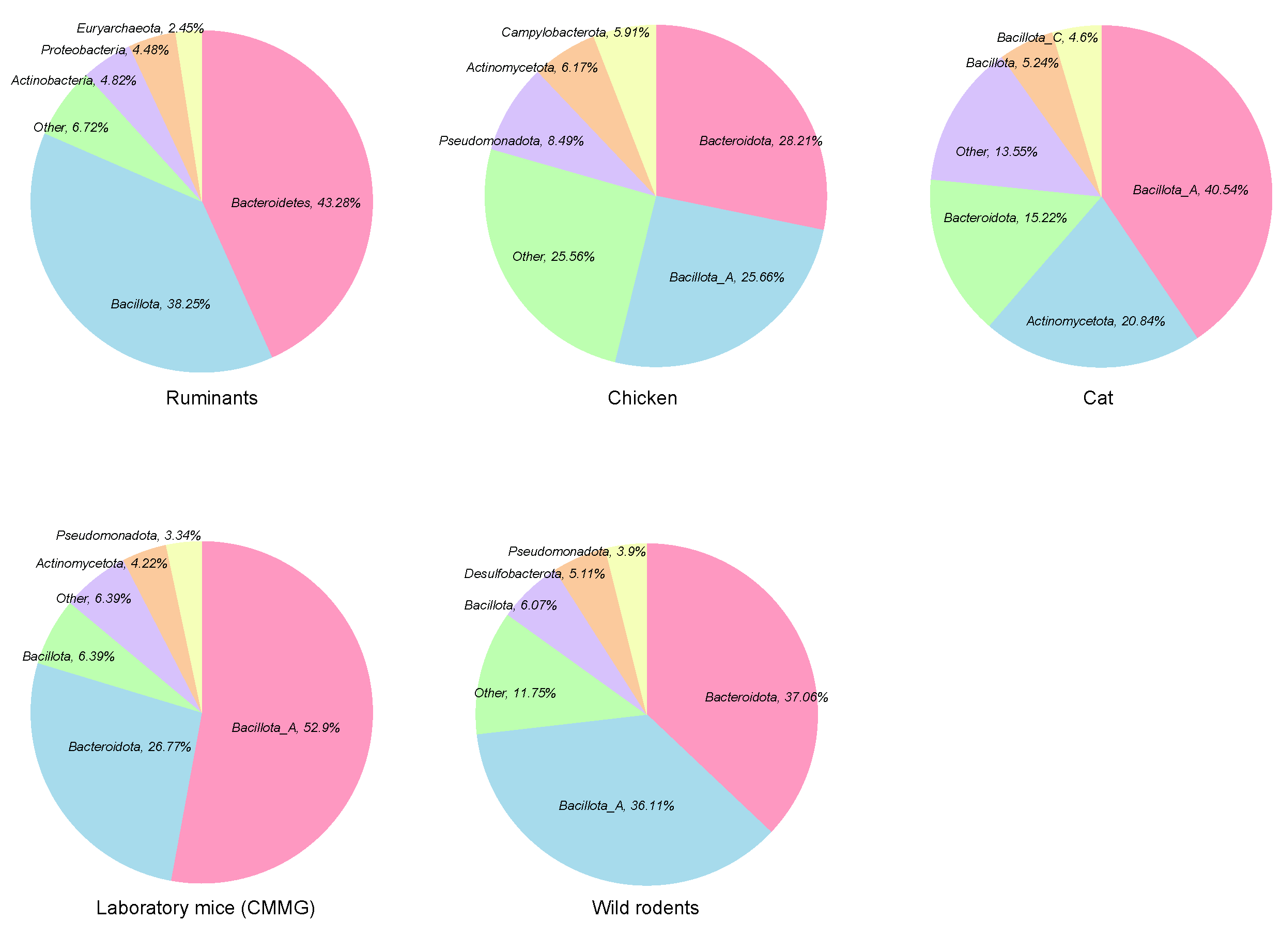


**Supplementary Figure 4.** **Proportion of genomes capable of *de novo* vitamin synthesis across animals.** The pie chart illustrates the proportion of genomes capable of synthesizing vitamins *de novo*, categorized by phylum-level classification across different animals.


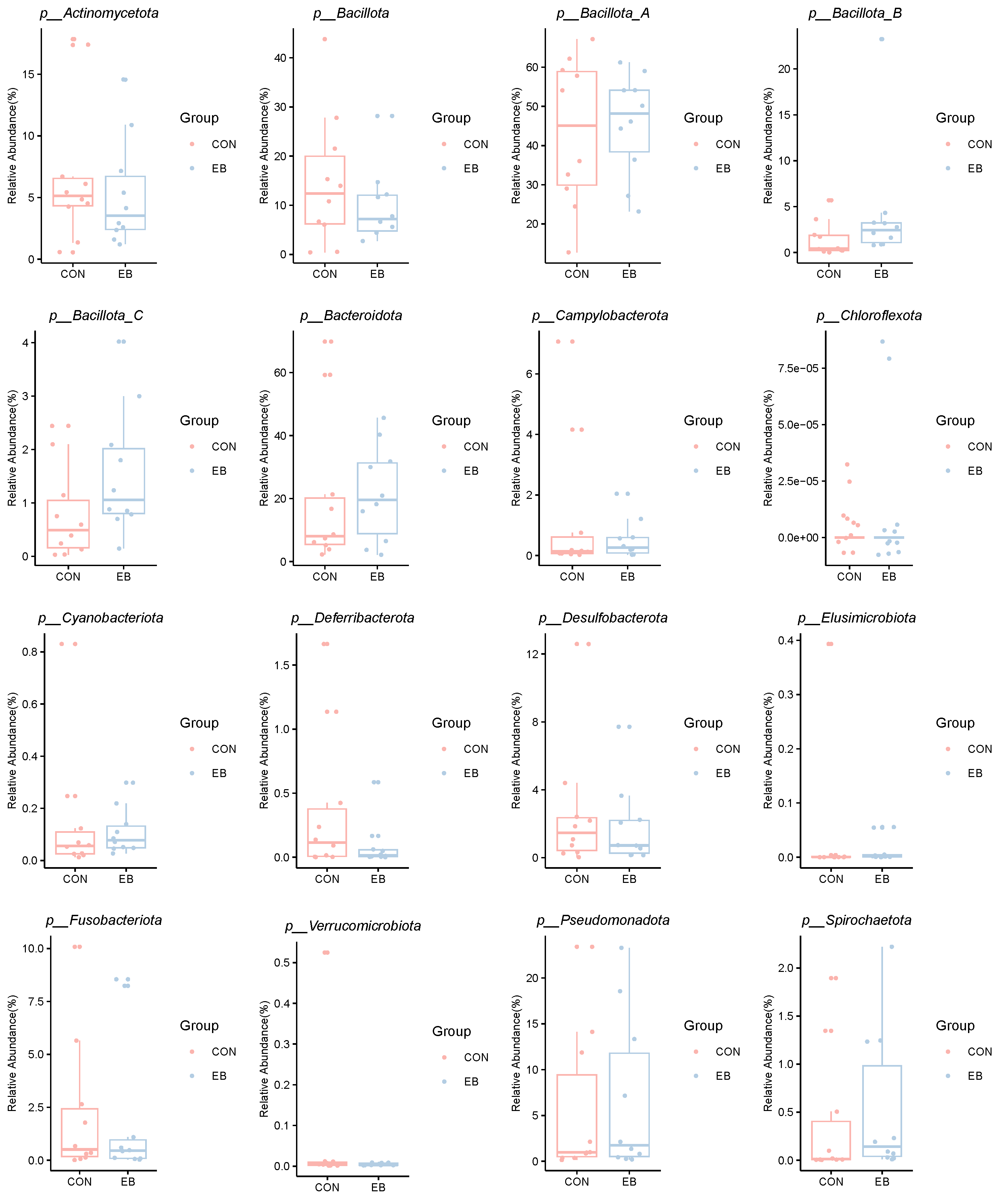


**Supplementary Figure 5.** **Changes in species abundance at the phylum level before and after microsporidium infection.** Box plots display differences in the relative abundance of species at the phylum level before and after *E. bieneusi* infection (excluding *Methanobacteriota*). Statistical significance was assessed using the Wilcoxon rank sum test (**p* < 0.05).

**Supplementary Tables**

**Supplementary Table 1.** Information on 20 wild rodent samples.

**Supplementary Table 2.** Summary of 9,929 representative genomes, including their sources, quality assessment criteria, and key characteristics.

**Supplementary Table 3.** Detailed overview of genes involved in the biosynthesis of vitamins B and K_2_, including relevant characteristics.

**Supplementary Table 4.** Taxonomic classification of 3,522 high-quality genomes, including their respective categories and levels.

**Supplementary Table 5.** Genomic statistics and vitamin synthesis capabilities of 2,307 selected genomes, highlighting key features and biosynthetic potential.

**Supplementary Table 6.** Relative abundances of functional roles involved in the pyridoxine biosynthesis pathway.
