# Supplementary material for: Gut microbiota response to Enterocytozoon bieneusi infection in wild rodents: enhanced vitamin B and K2 biosynthesis pathways
Source: BMC Genomics. 2026 Feb 5;27:258. doi: 10.1186/s12864-026-12575-4 (PMC12973552; doi:10.1186/s12864-026-12575-4)
Supplement: Supplementary file 1 — Supplementary Material 1. [file 12864_2026_12575_MOESM1_ESM.docx]

Supplementary Information

Gut microbiota response to Enterocytozoon bieneusi infection in wild rodents: enhanced vitamin B and K2 pathways

Xiao-Xuan Zhang1a, He Zhang2a, Ji-Xin Zhao1, Hai-Long Yu1,3, Chun-Ren Wang4, Kai-Meng Shang1, Yong-Jie Wei1, Ya Qin1,3, Jian-Ming Li5, Zi-Yu Zhao3, Chang-You Xia2*, Bei-Ni Chen6, Hany M. Elsheikha7* & He Ma1*

1 College of Veterinary Medicine, Qingdao Agricultural University, Qingdao, Shandong Province, PR China.
2 State Key Laboratory for Animal Disease Control and Prevention, Harbin Veterinary Research Institute, Chinese Academy of Agricultural Sciences, Harbin, Heilongjiang Province, PR China.
3 College of Veterinary Medicine, Jilin Agricultural University, Changchun, Jilin Province, PR China.
4 College of Animal Science and Veterinary Medicine, Heilongjiang Bayi Agricultural University, Daqing, Heilongjiang Province, PR China.
5 College of Chinese Medicinal Materials, Jilin Agricultural University, Changchun, Jilin Province, PR China; Jilin Provincial Engineering Research Center for Efficient Breeding and Product Development of Sika Deer, Changchun, Jilin Province, PR China; Key Laboratory of Animal Production and Product Quality and Security, Ministry of Education, Ministry of National Education, Changchun, Jilin Province, PR China.
6 College of Life Sciences, Changchun Sci-Tech University, Shuangyang, Jilin Province, PR China.
7 Faculty of Medicine and Health Sciences, School of Veterinary Medicine and Science, University of Nottingham, Sutton Bonington Campus, Loughborough, United Kingdom.

a These authors contributed equally.
*E-mail: xiachangyou@caas.cn (Chang-You Xia); Hany.Elsheikha@nottingham.ac.uk (Hany M. Elsheikha); mahe@qau.edu.cn (He Ma)

Supplementary Figures

Supplementary Figure 1. (A–C) Biosynthetic pathways of thiamine, niacin, and cobalamin. Functional roles are represented by rectangles, while metabolites are depicted as circles. 

Supplementary Figure 2. (A–C) Biosynthetic pathways of biotin, folate, and menaquinone. Functional roles are represented by rectangles, while metabolites are depicted as circles.


Supplementary Figure 3. (A–C) Biosynthetic pathways of pantothenate, pyridoxine, and riboflavin. Functional roles are represented by rectangles, while metabolites are depicted as circles.


Supplementary Figure 4. Proportion of genomes capable of de novo vitamin synthesis across animals. The pie chart illustrates the proportion of genomes capable of synthesizing vitamins de novo, categorized by phylum-level classification across different animals.


Supplementary Figure 5. Changes in species abundance at the phylum level before and after microsporidium infection. Box plots display differences in the relative abundance of species at the phylum level before and after E. bieneusi infection (excluding Methanobacteriota). Statistical significance was assessed using the Wilcoxon rank sum test (*p < 0.05).


Supplementary Tables
Supplementary Table 1. Information on 20 wild rodent samples.
Supplementary Table 2. Summary of 9,929 representative genomes, including their sources, quality assessment criteria, and key characteristics.
Supplementary Table 3. Detailed overview of genes involved in the biosynthesis of vitamins B and K2, including relevant characteristics.
Supplementary Table 4. Taxonomic classification of 3,522 high-quality genomes, including their respective categories and levels.
Supplementary Table 5. Genomic statistics and vitamin synthesis capabilities of 2,307 selected genomes, highlighting key features and biosynthetic potential.
Supplementary Table 6. Relative abundances of functional roles involved in the pyridoxine biosynthesis pathway.
